# The governance structure for data access in the DIRECT consortium: an innovative medicines initiative (IMI) project

**DOI:** 10.1186/s40504-018-0083-0

**Published:** 2018-09-04

**Authors:** Harriet J. A. Teare, Federico de Masi, Karina Banasik, Anna Barnett, Sanna Herrgard, Bernd Jablonka, Jacqueline W. M. Postma, Timothy J. McDonald, Ian Forgie, Piotr J. Chmura, Emil K. Rydzka, Ramneek Gupta, Soren Brunak, Ewan Pearson, Jane Kaye

**Affiliations:** 1 HeLEX Centre, University of Oxford, Ewert House, Banbury Road, Oxford, OX2 7DD UK; 2 Melbourne Law School, University of Melbourne, 185 Pelham Street, Carlton, VIC 3053 Australia; 3 Center for Biological Sequence Analysis, Department of Bio and Health Informatics, Technical University of Denmark, Building 208, DK-2800 Lyngby, Denmark; 4 Translational Disease Systems Biology, NNF Center for Protein Research, University of Copenhagen, Faculty of Health and Medical Sciences, Blegdamsvej 3B, DK-2200 Copenhagen, Denmark; 5 Division of Molecular & Clinical Medicine, School of Medicine, University of Dundee, Ninewells Hospital & Medical School, Dundee, UK; 6 Sanofi-Aventis Deutschland GmbH, Industriepark Höchst, 65926 Frankfurt, Germany; 7 Clinical Research Centre, Lund University Diabetes Centre, Box 50332, SE-202 13 Malmö, Sweden; 8 Blood Sciences, Template A2, Royal Devon and Exeter Hospital, Barrack Road, Exeter, EX2 5DW UK

**Keywords:** IMI project, Governance, Data access, Data access committee

## Abstract

Biomedical research projects involving multiple partners from public and private sectors require coherent internal governance mechanisms to engender good working relationships. The DIRECT project is an example of such a venture, funded by the Innovative Medicines Initiative Joint Undertaking (IMI JU). This paper describes the data access policy that was developed within DIRECT to support data access and sharing, via the establishment of a 3-tiered Data Access Committee. The process was intended to allow quick access to data, whilst enabling strong oversight of how data were being accessed and by whom, and any subsequent analyses, to contribute to the overall objectives of the consortium.

## Introduction

Biomedical research is increasingly carried out by large research consortia, which bring together multi-disciplinary teams, funded to focus on specific research challenges. (Disis & Slattery, 2010; Goldman, 2012) The complexity of the research activities amongst these partners, including the processing and sharing of samples and data is often supported by the development of an internal governance framework. The DIRECT (DIabetes REsearCh on patient stratification) project, (http://www.imi.europa.eu/projects-results/project-factsheets/direct) which focuses on identifying biomarkers for improved diabetes patient stratification, funded under the Innovative Medicines Initiative Joint Undertaking (IMI JU) is an example of such a consortium. This Initiative is designed to bring commercial and academic partners together to share expertise and resources to accelerate health innovation (http://www.imi.europa.eu/about-imi/mission-objectives). IMI considers that a more focused collaboration between academia, small and medium-sized enterprises and the pharmaceutical industry will solve bottlenecks in development and enable potential treatments and therapies to move to clinical trials more quickly. The success of the endeavour therefore relies on good working relationships between partners and aligning different motivations to achieve shared goals and cultivate responsible innovation. One of the mechanisms to foster good working relationships is to establish transparent governance mechanisms that will underpin the collaboration, and instil a high degree of accountability in all partners. Governance mechanisms for the appropriate and lawful use of patient data are a prime requirement to address the challenges of working across borders and sectors. The consortium must comply with data management and ethical approval processes within each participating country. Equally importantly, the consortium is responsible to research participants, to ensure the use of personal data and samples meets their expectations and the conditions established in the consent agreements – both in secure handling of their data, and in ensuring that their data is used efficiently to combat diabetes. The use of the data may also be influenced by the funding body’s policies and its agreements with project partners, in accordance with the objectives of the project. Establishing clear processes and policies that set out how these different interests will be recognised and supported within the consortium thus underpins the research endeavour and directly supports scientific progress.

The aims of DIRECT are ‘to identify biomarkers that address current bottlenecks in diabetes drug development and to develop a stratified medicines approach to the treatment of type 2 diabetes with either existing or novel therapies. The identification and validation of important biomarkers for glycaemic deterioration and treatment response may be used to predict and monitor the effect of therapeutic interventions in subtypes of diabetes with different pathophysiology’ (http://www.direct-diabetes.org/objectives/). This is of interest from an academic perspective, in furthering knowledge about disease progression, and may be of commercial interest to industry partners if it leads to opportunities to decrease size and length of clinical trials and thus speed up introduction of new diagnostics, prevention and treatment. To achieve these ends, we have created a dataset of clinical, genotypic and other omics data to analyse for patient subgroup-specific biomarkers, which we must process and share securely to comply with legal and ethical requirements and to maintain clinical trial subject confidence.

Legal requirements include the GDPR (and national implementations), the Act of Health Ethics (http://www.nvk.dk/english/act-on-research) including the conditions laid down by the National Committee on Health Research Ethics, and the Danish Code of Conduct for research Integrity. The governance structure for data management in the DIRECT consortium was designed to fulfill these legal requirements and the trial subjects’ sense of trust in the consortium. However, it was also important that there would be quick access to data to support the intended research. The objective of the consortium is of importance here: the data subjects are patients suffering from the disease in question and the research purpose is the production of knowledge that will be relevant to the patient group. Maximizing the usefulness of the data given by the patients, by making data access procedures effective, is in the patients’ interests and points the governance structure in the direction of agility.

While the regulatory and health service environment provides guidance on requirements such as obtaining ethical approval and how to handle data in a lawful manner, (http://www.hra.nhs.uk/, https://www.hra.nhs.uk/hra-guidance-general-data-protection-regulation/) it is largely silent on governance procedures and policies that should be put in place for consortia. For this reason, there have been a number of publications outlining governance structures within large consortia, such as those adopted in UK10K (Rare Genetic Variants in Health and Disease), (Muddyman et al., 2013) the ICGC (International Cancer Genome Consortium) (Joly et al., 2012) and ENGAGE (European Network for Genetic and Genomic Epidemiology). (Budin-Ljøsne et al., 2014) International guidelines for data sharing and access, for example from the OECD, (http://www.oecd.org/science/sci-tech/38500813.pdf) EAGDA, (https://wellcome.ac.uk/sites/default/files/data-management-plans.pdf) and the increasing focus on FAIR principles of data sharing (findable, accessible, interoperable, reusable) (Wilkinson et al., 2016) provide important insight surrounding data sharing between projects. However, while providing for shareability of data gathered from different sources (for example by using CRFs, Case Report Forms), these guidelines do not attend to the specifics for sharing of both pre-existing and newly collected data within projects. The situation where commercial and academic partners work together is even less well described: to our knowledge, there has only been one other paper outlining the governance system required for an IMI project. (Morrison et al., 2015) By their nature, these require commercial and academic partners to work together and, therefore, have an additional layer of complexity relating to the use of data and samples. In 2008, the European Commission and industrial partners committed a billion Euros each to support new initiatives in health. The aim of this funding was to accelerate innovation by bringing together commercial and academic partners. Typically, researchers are based across Europe and work together in interdisciplinary teams in an IMI project, to focus on specific research questions. IMI JU Funding therefore requires collaboration both across sectors but also across disciplines and geographical boundaries (Innovative Medicines Initiative, 2018).

The purpose of this paper is to outline the internal governance system that was developed in the DIRECT project to support the use of samples and data between the project partners, and to discuss some of the challenges of establishing such structures.

### Designing a data access system

The success of the DIRECT project relied specifically on the accumulation of complex data, with major scientific advances likely to emanate from the analyses of combined datasets by multi-disciplinary teams. Large, existing datasets were brought into the project and others were generated as the project progressed. The datasets developed through DIRECT had different formats originating from sample analysis at a variety of laboratories and clinical studies conducted at multiple centres across Europe. The DIRECT work packages were tightly interlinked, with results from one study contributing directly to the planning and/or analyses of others.

**Fig. 1:**
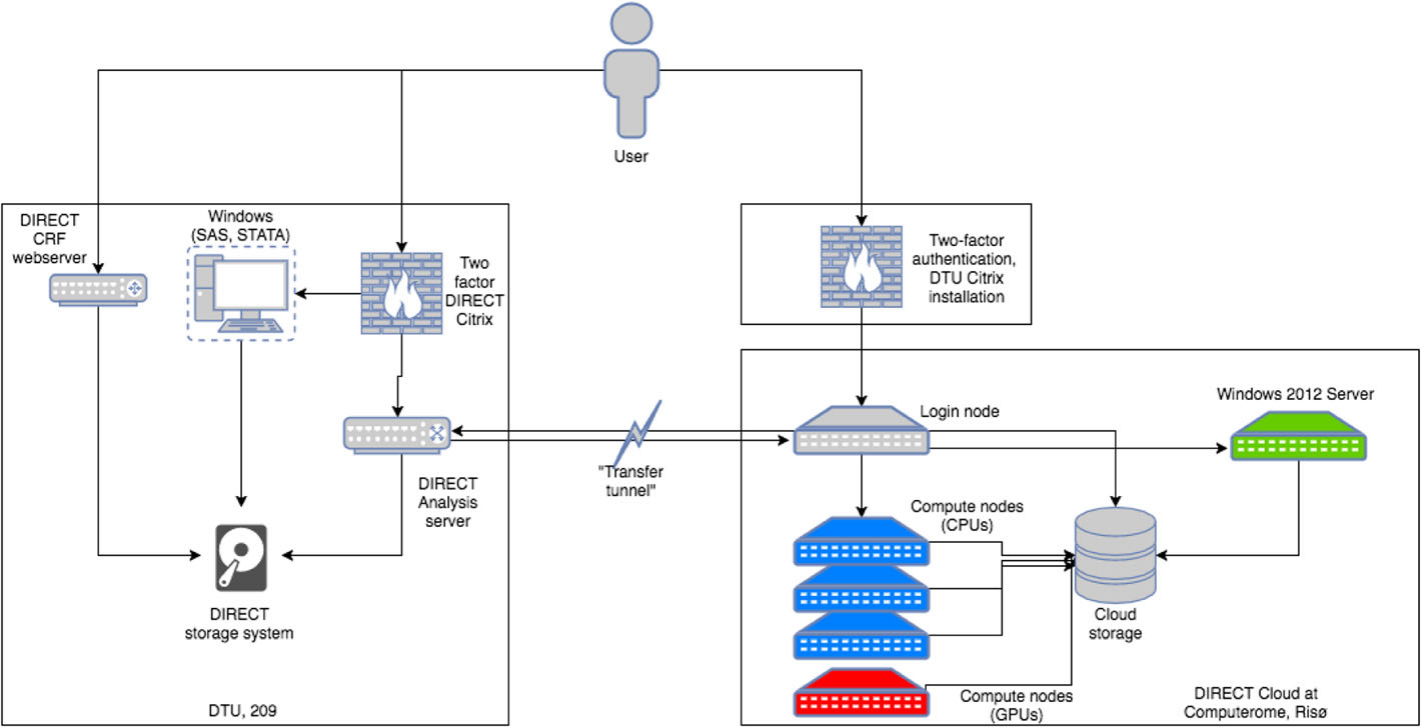
The DIRECT Database Server is stored in a room with restricted access and added protection against physical intrusion. The access for data entry is possible only through a secure, encrypted protocol between the server and the Data Entry Clients (https). Only users cleared for data entry can access this functionality by username and password. The DIRECT Analysis Secure Private Cloud is a composition of: A UNIX server (OpenSuSE 12.2) equipped with a standard set of UNIX tools. The DIRECT Analysis server is located in the same room as the DIRECT Database server, and, thus, has the same physical security measures. A UNIX cloud (CentOS7) composed of a login node and a variable number of computational nodes onto which jobs are distributed using MOAB queuing system. Each node consists of 28 CPUs and 128GB RAM. Additionally, a node containing 2 nVidia Tesla P100 GPU cards is accessible for highly computational requiring analyses, such as neural networks and machine learning algorithms. Access to the Analysis Cloud was limited to named individuals approved by the DAC. Access to the Analysis Cloud went through a two-factor CITRIX installation with SMS passcode (authentication was done through a password and a one-time passcode sent through an SMS). All communication between the user and the CITRIX system was through an SSL connection with 128-bit encryption. No data could be exported from or uploaded to the Analysis Cloud through the CITRIX installation

Essential to DIRECT was a custom-built centralised database (The DIRECT Database Server) that required a controlled-access data-hosting environment to ensure extremely high levels of data security. All data contributed to, or generated within DIRECT, were uploaded onto the database to provide central and secure data storage. Data analyses were carried out on a separate secure server, the DIRECT Analysis Secure Private Cloud. (The Secure Private Cloud – Computerome Secure Private Cloud service – that DIRECT used to build its analysis environment provides the flexibility of a public cloud model, but does not introduce the critical issue of sharing physical infrastructure with others. This is the key difference between the Computerome and similar systems across the Europe) (Fig. 1 Secure cloud infrastructure) Statistical and other necessary software were installed as needed to facilitate data analyses on the server and to severely limit (preferably avoid) the need to download data from the server onto potentially less secure platforms. This set-up led to one of the discernible challenges in the DIRECT project: how to develop an internal governance system to share samples and data (which would have looked very different if a federated structure had been used instead)? (Muilu et al., 2007).

Timely access to data was a key factor in determining whether milestones could be met and deliverables generated across the project. It was, therefore, necessary to devise an agile system for data access that would not impose lengthy bureaucracy, would comply with agreed consortium policies and provide reassurance that all requests were carefully evaluated within the context of work packages and the project as a whole, which was also responsive to the changing needs of the consortium.

The data held within DIRECT can be split into two groups: retrospective data, brought into the project by consortium members (e.g. generated from previous projects), and prospective data generated over the course of DIRECT. When planning how to manage retrospective data, it was necessary to meet previous conditions such as timelines, preferential use, scope of analysis, and any consent restrictions linked with these datasets. Retrospective data was supplied by researchers that had either been involved directly in gathering the data, or in the projects that had first used it. They were familiar with the context under which it had been collected, and the initial ethical processes the projects had met to allow the data to be gathered. This knowledge was brought into DIRECT along with the data and recognised when constructing the data access policy. Each DIRECT study was approved through local ethics committees that considered use of both retrospective and prospective data, and the appropriateness of the studies against the original purposes for which the data had been gathered. The Management Board provided further oversight for consideration of data use, and where necessary referred to the consortium partners that had contributed retrospective data to confirm that DIRECT plans were in line with the intended use of the data, on behalf of the participants from whom it was derived. Furthermore, there were differences between the datasets such as phenotype definitions used, data units, or methods for collecting data, all of which could significantly hinder data harmonisation. Prospective data, on the other hand, were generated across the consortium; these needed to be equally available to all members.

It was anticipated that requests for access to data would fall into two broad categories: those that were within the scope of the central research objectives for DIRECT, and those that were outside the scope of the DIRECT project. While questions within scope needed to take priority, it was important to ensure that the rich datasets generated within DIRECT could be used to their full potential (provided use was in accordance with participants’ consent, and did not undermine or conflict with the DIRECT objectives). Therefore, the system implemented should not discourage ancillary projects or tangential research. To date, all data access requests received during the project have been for analyses directly relevant to, and therefore within scope of, DIRECT. The procedures for accessing data had to be quick, but also enable fair and transparent access for all consortium members, while maintaining oversight of how data were being used throughout the project, and meeting the needs and expectations of individual partners as well as the funding body.

To design the governance structure and the policy and procedures to implement them, a specialist committee was set up by the Management Board and was directly accountable to it. The Sample and Data Committee (SDC) was tasked to develop all policy relevant to DIRECT internal governance, including data quality and publications, as well as data access. The committee included representation from the project management, ethics, law and regulation, database and central laboratory teams, as well as researchers with experience working in other major consortia, including other IMI JU funded projects, like SUMMIT. (SUMMIT, 2018) The data access policy was developed through discussion and consensus-building and drew upon input from experts across the consortium as required, so that the policy for accessing data considered the needs of all researchers and the types of research within DIRECT. The SDC having reached consensus, presented the data access policy to the Management Board and finally to the DIRECT plenum for consultation. The final policy was reviewed and approved by the Management Board. After launch, the data access policy was reviewed on a “fit for purpose” basis with opportunity to revise and adapt it, as required.

### Data access committee

A Data Access Committee (DAC) was set up to manage access to DIRECT data. This committee consisted of three tiers, depending on the level of consideration required for a specific request. The three-tiered structure of the DAC enabled requests from DIRECT partners to be assessed and processed quickly and efficiently. An executive (DAC Executive Group, ‘Tier 1’) was introduced to implement a triage system, to allow for quick consideration of all requests, and immediate approval of straightforward requests, that clearly fitted within the scope of DIRECT. More complicated requests would be referred to the full DAC (‘Tier 2’, made up of work package leads) and, finally, to the wider Management Board (‘Tier 3’). There was overlap of personnel between the SDC and the DAC Executive Group, so that policy writers were familiar with implementation efforts and the practicalities of policy application. The DAC executive group included representation from the database team, the project management team and ethical and legal expertise, and is separate from the Management Board. Thus, any scenarios that fell outside of current policy, yet needed to be covered, could be quickly identified and dovetailed with any requirement for policy change. An example of this is provided below, when considering the technical considerations for implementation, which identified practical limitations of the set-up, which subsequently led to a revision of the policy.

**Table:** Box 1 The data access committee tier structure

Tier 1: The Executive Group was responsible for collecting Data Access Forms (DAF) and triaging initial requests for access to DIRECT data. In instances where access was approved, the Executive Group notified the requestor of the decision and forwarded the request to the database team to initiate access to the required data. The Executive Group, comprising 3 people, could approve straightforward applications that were clearly within the scope of the DIRECT project, and would refer more complicated requests to the wider DAC. The Executive Group, therefore, could decide whether the request was 1) approved, 2) referred to the DAC or 3) referred to the Data Provider(s) and sample Custodian(s) by a majority agreement of 2/3. Agreement had to be reached within a stated deadline.	
Tier 2: The wider DAC, constituting Work Package (WP) leads, was responsible for reviewing access requests that had been referred from the Executive Group. This process included confirming whether a request fell outside the scope of the DIRECT project. A decision required 5 of the 7-strong committee to respond and had to be reached within a stated deadline.	
Tier 3: The Management Board If the DAC could not reach a decision, or if there was a difference of opinion between the requesting researcher and WP leads, the matter would be referred to the full Management Board for adjudication. If there were still areas of dispute, the funders would be called upon to provide a final opinion.	

### The triage process

All researchers wanting to access data stored on the DIRECT database needed to complete a Data Access Form (DAF). This form had two purposes: Firstly, it identified the research question to be addressed and secondly, it enabled researchers to stipulate exactly to which data they required access and the names and institutional affiliation of the investigators who required access. (Additional names could be added to – or deleted from – the Data Access Format at a later date as required). The electronic form was simple to complete and used ‘drop-down’ menu options wherever possible. The DAC Executive Group provided a streamlined process for data provision, making it quicker and easier for researchers to access the data they needed. These transparent and accountable management structures also ensured that the project conformed to legal requirements and good information governance standards.

**Fig. 2:**
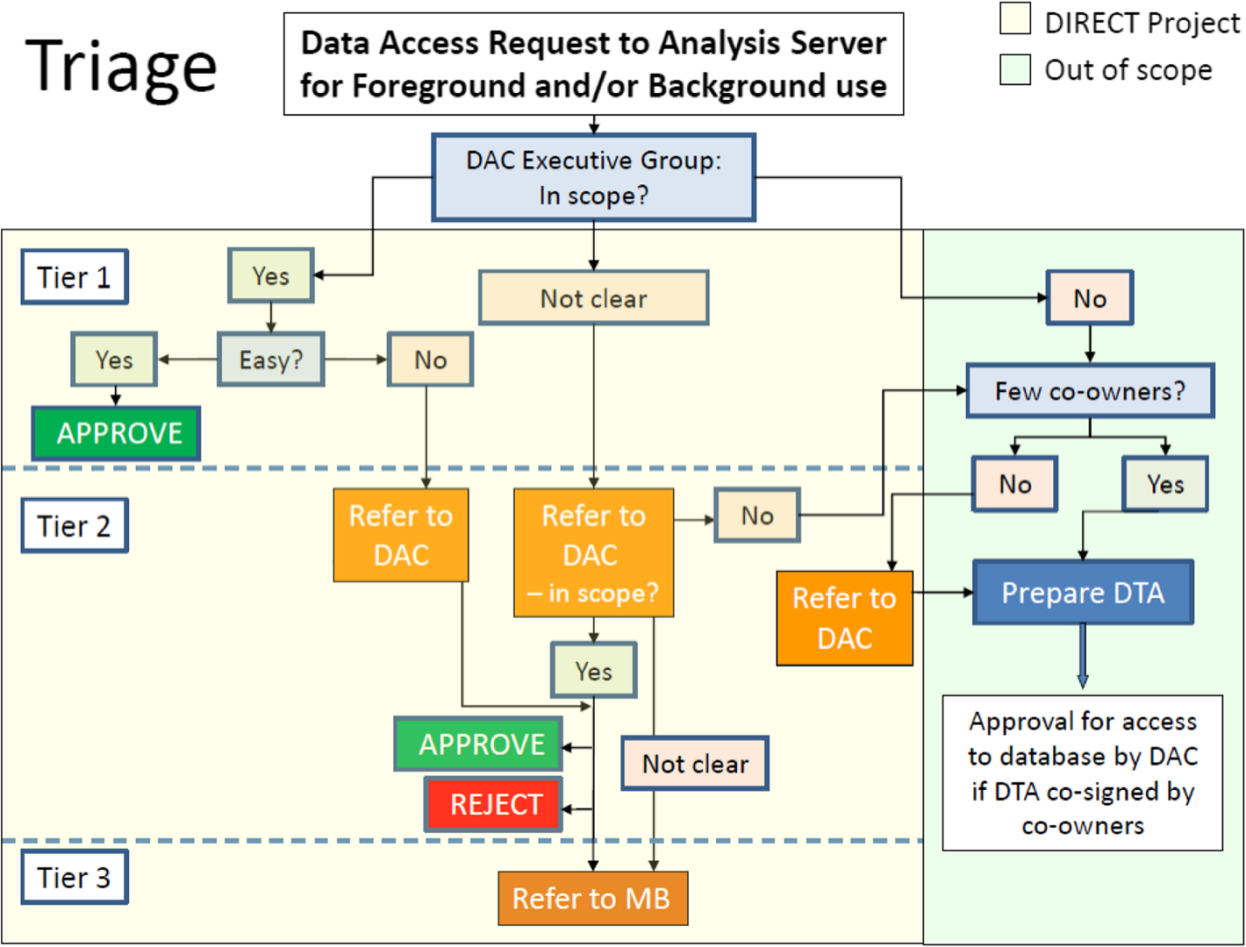
The DAC triage process for data access requests. DAC: Data Access Committee. DTA: Data Transfer Agreement. In scope? Is the request clearly within the remit of DIRECT?. Easy? Is the request uncontentious, clearly linked to a specific dataset and work package, timely, with a robust research question and analysis plan, and in accordance with ethical, legal and regulatory (ELR) requirements?. Few Co-owners? Does the request correspond to a single owner or small group of owners, or does it affect a large group (e.g. whole consortium)? In instances where a large group needs to be consulted, the DAC may need to consider to whom to refer the request
  Once a request had been approved, time-bound access to the DIRECT Analysis Server was granted. This enabled access to the individual-level data, with each researcher/work package analysis team able to access their own area or a shared area for performing data analysis.

Two types of request were received:

1. Researchers accessing their own data:
  A central principle within the project was that all researchers would be able to access their own data via the Analysis Server, or as a download from the DIRECT database. This was an important incentive to encourage researchers to upload centre data in a timely fashion and to make it available to the consortium. While researchers wishing to access their own data were still required to submit a DAF, this was to facilitate the database team in providing access to the required data, and to maintain oversight of who was doing what. Requests for access to centres’ own data were automatically considered to be ‘uncontentious’ and approved by the Tier 1 DAC Executive Group immediately.
2. Researchers accessing composite data
  For researchers wanting to access a composite dataset, which could include data that they had uploaded (own data), sample analysis by other research groups (which had co-ownership), and/or included other centres’ data, a data request was submitted, which stipulated the research question, the data requested and included a plan and timeline for the intended analysis. The DAF was submitted to the DAC Executive Group, who considered the request, and either approved it, if it was in scope and adhered to a set of conditions (Table 1), or referred it to the wider DAC if it was unclear (the triage process, see Fig. 2: The DAC triage process for data access requests). A separate DAF was required for each separate research question or request for access. An Analysis Plan was an important part of the application for accessing data, to ensure that researchers had fully considered the analysis that they wanted to conduct and could demonstrate how this plan was in-line with the scope of the project. Each Analysis Plan had to have been discussed with and approved by the work package lead, and submitted as part of the access request, as the DAC was not responsible for evaluating and approving scientific content.
  Each approved DAF authorised access to particular portions of the data. The data itself was divided into different topics or themes, informed by the different Analysis Plans that were generated and these in turn resulted in the creation of a team (from team01 to teamXX) and a relevant “teamXX” folder into which the requested data were made available. If teams required access to additional data, a DAF was submitted, and if approved, the resulting data would be added to the pre-existing team folder. eCRF, clinical and omics data were extracted from the relevant database tables and server locations and delivered to team analysts, either as CSV files or simlinks to the securely stored datasets.

**Table 1:** Conditions for approval

- Timely - Enabling collaboration - Indicated scientist(s) who would do the analysis - Indicated specific data requested for analysis - Outlined a robust research question and an analysis plan - Outlined a robust method for analysis - Was not in breach of any ethical, legal or regulatory requirements - Fitted clearly within a specific work package

### Accessing DIRECT data for research outside the scope of DIRECT

**Fig. 3:**
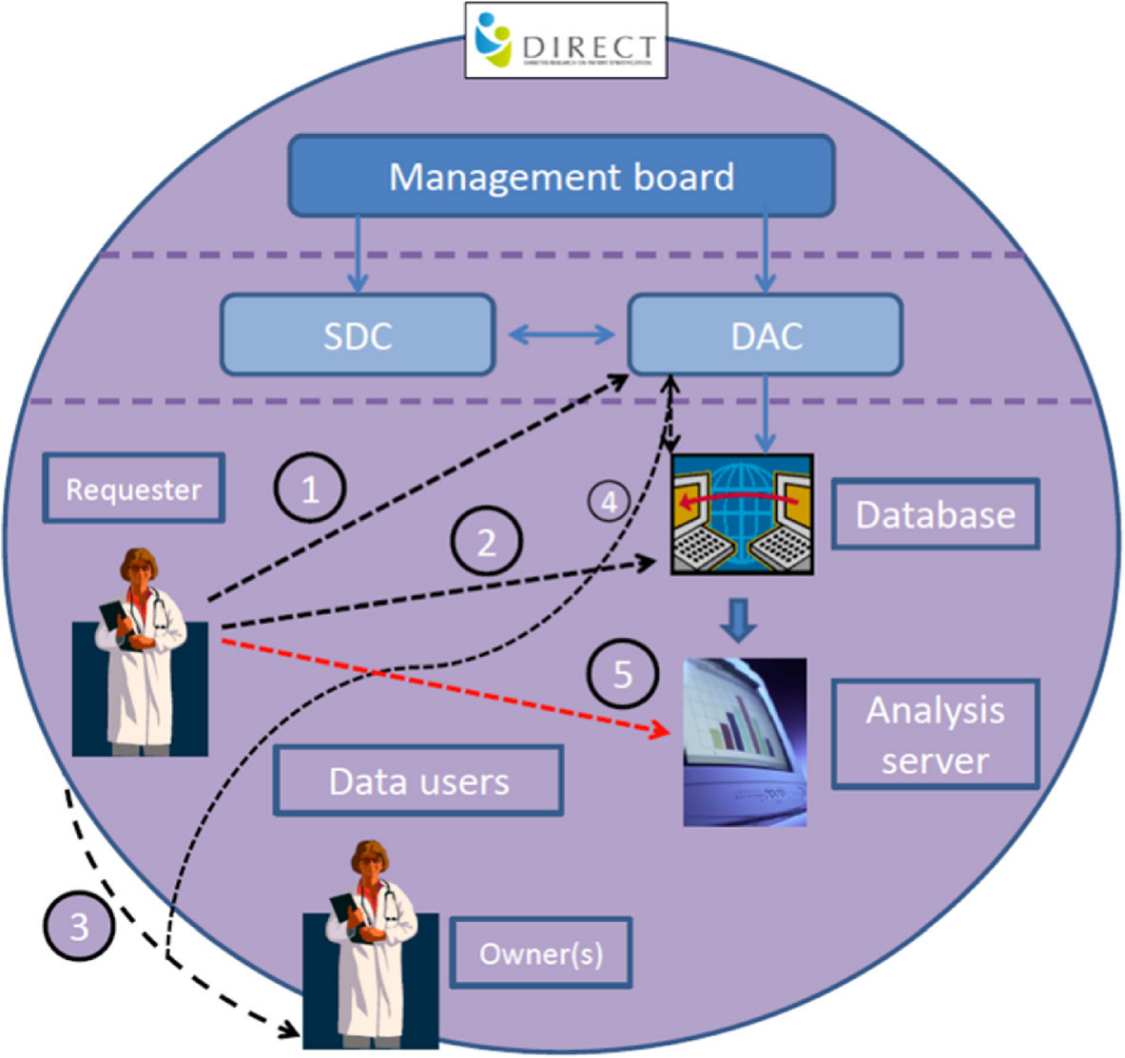
Data Access within DIRECT. 1: Data Access Form (DAF) – to be completed and submitted to the DAC to request access to data from the database. 2: Data Transfer Agreement (DTA) – to be completed by the requesting researcher and the data owners to transfer data for research outside the scope of DIRECT. 3: All DTAs must be sent to the DAC to notify of data sharing outside the database and will be forwarded to the DTU if access to the database is required. 4: If the DAC approves access to data, the data is made available to the requester(s) on the analysis server

If the DAC considered a research question to be outside the scope of DIRECT, the DAF would be returned to the data requester. Should the requestor still wish to access the required data, the requester would then contact the recognised owner of the data (the team from which the data have been contributed or collected) to draw up a Data Transfer Agreement (DTA), to be agreed and co-signed by the owner(s) of the requested data and approved by the legal representatives of the institutes involved. The agreement would identify which data were to be shared, and the conditions for transfer. (Figure 3: Data Access within DIRECT).

While this process was set-up to involve the owners of the data, as a separate agreement outside the DIRECT project, it was agreed that access could be provided via the DIRECT Analysis Secure Private Cloud, to encourage and facilitate further use of the rich datasets collected within DIRECT.

If the data were transferred directly from the data owner(s) to the data receiver (thus bypassing the DIRECT data storage infrastructure), the owner(s) and receiver were responsible for ensuring the security of the data, and that it would be used in accordance with all ethical, legal and regulatory requirements and had institutional approval.

### External requests for access to DIRECT data

When creating a rich, multi-centre database, requests for access to data from researchers outside the consortium are to be anticipated. Whereas the agreement between the DIRECT partners, as well as the IMI-JU IP Policy, provides regulation of third party data access for the period following the completion of the project, such a delay in accessibility may not be in the best interest of either research progress or the patients’ health. While project members were committed to considering ways to maximise use of the rich dataset produced within DIRECT, sharing data had to be balanced against control of data use as assured in the informed consent processes, as well as potentially compromising the consortium’s own objectives. The conclusion was that external requests for access would be evaluated on a case-by-case basis by the Management Board and then shared with all partners within the consortium. In case the request involved a discrete dataset, it could be considered to set up a collaboration agreement between the external requester and relevant DIRECT researchers, with the latter performing analyses in the database and sharing the results with the external researcher, thus avoiding the need for data to travel outside of the DIRECT project. Requests for larger datasets, or access to the entire database would require more significant consideration. Within DIRECT, a qualitative study was conducted which surveyed participants on their views about sharing data beyond the project (Shah, 2018). The findings of this study will inform a more comprehensive data sharing policy that will be developed ahead of the project conclusion.

### Technical considerations

In an ideal situation, access would be given only to required data, and then to updates of the data as they were available. However, it was quickly realised that this would be unrealistic from a technical perspective. Providing such granular access would put a heavy demand on the database team to identify, arrange and update the data. It would also have required strict oversight of updates to the database, to keep track of which researchers had access to which versions.

It was, therefore, initially decided that data would be partitioned and organised into relevant folders on the analysis server, and that requestors would be given access to the folders in which their requested data were stored. In the case of the DIRECT consortium, the manageable number of partition folders was defined as between 20 and 30, based on the number of work package tasks, with additional partitions to isolate sensitive or large data only needed for specific types of analysis, such as the storage of MRI scans. eCRF and clinical data would be extracted from the main database and delivered to analysts as data tables only, containing values for the specifically requested variables.

This approach made it easier for the database team to manage and update the system and ensured that analysis was being conducted on the most up-to-date dataset available and with controlled data access for each research team. Teams would get access only to the requested data, nothing more (Table 2).

**Table:** Box 3 Examples of access requests:

1: An analyst requesting insulin results received access to only 1 out of 25 folders containing biochemistry results for that particular study. In addition to insulin results, other clinical chemistry was accessible (but only for that study/task). 2: A team requesting access to dietary questionnaires and phenotype data received access to 1 out of 25 folders, containing the dietary questionnaires for a particular study and one set of csv files containing the requested clinical/eCRF data variables.

**Table 2:** Data organisation (non - eCRF)

	Accelerometer	Diet	Genomics	Glucagon	GLP-1	Glucose Lowering	Glycemic Modeling	Lipidomics	Metabolomics	Microbiome	MRI Derived	MRI Raw	ProInsulin	Prospective Metabolomics	Proteomics	Targeted Metabolomics	Transcriptomics
WP2.1	X	X	X	X	X	Drugs as Covariates	X		X	X			X		X	X	
WP2.2	X	X	X	X	X		X		X	X			X		X	X	
WP3.1																	
WP3.2															X	X	
WP3.3								X	X								
WP3.4																	
WP3.5									X					X			
WP3																	
WP2									X								X

Subsequently, this system has been revised; a system of “team folders” was implemented that contained data relevant to certain research questions, such as diabetes progression or omics analysis. In some instances, it became necessary for some analysts to be provided with access to multiple team folders to perform the analyses they were undertaking. However, the system ensured that this was both documented and transparent.

One specific challenge encountered, as more data were uploaded onto the server, was how to communicate what was available for analysis. To address this, a data warehouse catalogue was proposed to provide a map of the database. Even so, there were instances where researchers requested access to specific datasets to establish the demographic of participants for whom data were available, to assist with planning of analysis. These requests were handled on a case-by-case basis and demonstrated the value of having a DAC that was well-acquainted with the different work packages and the sort of research that was being conducted across the project.

### Challenges of implementation

Designing an internal governance structure to oversee access to a centralised database for research purposes can be a time consuming and politically sensitive process. Such a research consortium brings together experts each with different backgrounds and possibly also previous experiences that may influence their expectations and requirements for taking part. The perennial issues with research, such as academic rivalries or worries of being scooped, and, on the technical level, the use of different formats and metrics between the commercial and academic partners have been well documented elsewhere, (Borgman, 2012) needed to be taken into consideration in developing a policy that was intended to facilitate consortium-wide data access.

Added to this is the challenge of working across international borders, where data protection requirements can vary, and indeed where data protection legislation has changed during the project, e.g. with the recent introduction of the General Data Protection Regulation (GDPR). The central database is hosted in Denmark, which traditionally has very strict requirements relating to data security and handling.

Participants in each recruiting country were briefed on how data would be stored and shared, and this information provision was validated through local ethics approvals. As held in *Breyer (2016) (C-582/14),* for information to be treated as personal data there is no requirement that all the information enabling the identification of the data subject be in the hands of one person; data is therefore treated as personal data even though the consortium partner in Denmark, in charge of the database, has no means of identifying the natural persons whose data is stored.

There will be different views across a consortium about the process for access and whether it is even necessary. For example, researchers that are responsible for the generation of primary data may consider oversight and management to be very important to ensure that data are not exploited in an uncontrolled way. However, data analysts may be more interested in the speed by which they can access the data and less keen on having a complicated bureaucratic process.(Kaye et al., 2009) Getting access to the full width of a rich database for a broad exploration, without explicit hypotheses, may be appealing from a scientific or commercial point of view but can be difficult to align with the GDPR demands on supplying a patient with information about the use of personal data. Striking a balance between these seemingly opposing views is important for the consortium to run smoothly.

As part of the project Grant Agreement, all data producers were required to make their results available to the consortium, but this raised issues on who should have access to data and who should be recognised for data generation. More importantly, checks needed to be put in place to ensure that proposed DIRECT research fitted with the consent given by study subjects and was also aligned to the objectives of the study/project, as set out in the project Description of Work, from which they were derived. Previous experiences of research collaborations also greatly influence the expectations of researchers embarking on a new consortium project. Previous abuses of trust may be carried into new projects and influence the stipulations laid forth on how data are stored and shared, and who should have oversight of these processes.

Central to ensuring that data could be accessed in a timely manner was the system of triage, whereby the DAC Executive Group could consider and approve questions that clearly fell within the scope of DIRECT and responded to central questions within the project. By having a committee tasked with checking the content of requests, and ensuring all required information was included, any requests that did require referral to the wider DAC, and thus involved a larger number of people, did not take longer than necessary to reach a decision. The DAC Executive Group provided a useful point of contact for queries relating to specific aspects of access for data, which enabled many issues to be anticipated and discussed in advance of the data being required.

### Consultation with the consortium

Once the SDC had proposed this system to the Management Board, the data access policy was circulated to the Plenum for consultation. Feedback from this process was largely supportive from both academic and industry partners and did not lead to major amendments. (It is worth bearing in mind that the DIRECT project is pre-competitive and that access regulation in the Project Agreement distinguishes between data use for i) completing the project, ii) research use, iii) direct exploitation, in which data owners have no obligation to make data available in case iii). The main request resulting from this process was to include a sign-off procedure for participants to confirm that they had read and understood the consortium’s Data Access Policy and SOP and the terms and conditions for accessing data. This was deemed important to ensure that participants acknowledged their responsibilities for keeping data secure and using it appropriately, and reflected previous experiences of data misuse. Consequently, all requestors wishing access to data were required to sign the last page of the policy and send a scan of this to the DAC. Following approval of this procedure by the Management Board, the policy and associated procedures and forms were implemented within the consortium. Data access requests were submitted to the DAC as soon as the systems were in place, and this continued at a steady pace throughout the early stages of the project.

The application of a well-elaborated data access governance policy was also of high interest for participating industry partners. As defined in the Project Agreement signed by all DIRECT consortium members, consortium participants are granted royalty-free access to project data (retro- and prospective data), if the data are used for completing the project. Access rights to the data for the purpose of research use are granted on fair and reasonable terms. This governance structure for data access in the DIRECT consortium ensures a clear and efficient regulation of data access via the submission of data requests to the DAC, which is transparent to all consortium members and allows the discrimination of data access requests between project-related and research purposes.

### Limitations

The process for developing the data access arrangements relied on a small group of partners working on behalf of the consortium, representing both academia and industry, to devise a system that would be relevant to the entire project. The reason for creating a small group was the need to progress: devising this model of data access was one of the many priorities that arise at the start of a project of this scale. The consultation process, which included the entire consortium, was intended to allow all partners to contribute to the final processes for considering access to data. It was impossible to guarantee that everyone fully engaged with this process, and therefore fully supported the final iteration of the policy, yet we achieved a reasonable representation across the institutions represented within the group. Because it is always difficult to know if consensus has been actively reached, we emphasised active awareness-raising and information about the policy, to ensure all consortium members of its existence, what it covered, and what the expectations were for close collaboration. As indicated, this included requiring analysts requesting access to data to sign a form that confirmed that they had read and understood the consortium’s data access policy. Given there were multiple solutions for data access, ranging from everyone having access to everything, to a complete lock-down of data, the policy development was driven by enabling data security and the efficient use of data, striking a balance between control of the data and allowing research progress to be made, in accordance with the objectives of the consortium. Maximizing the use of existing research data avoids the need to repeat the same measurements over and over again, and avoiding patients being exposed to repeat sampling, which would be ethically questionable. This is reflected in the policy, and associated procedures. Anecdotal feedback from members of the consortium suggests that the policy was not too onerous, and that they understood the need for oversight and transparency in data use. To date over 60 requests have been made to the DAC, with almost all requests approved. The high success rate for approvals is partly due to the clear objectives within the study, and multiple opportunities for consortium members to discuss research plans together, and partly due to the additional step that requires an Analysis Plan regarding a particular theme of the project to be submitted to, and approved by, the Management Board, before a request for access can be made. This helps both ensure that we have a clear view of the data itself, and that high quality research is being conducted, that meets the overall objectives of the project.

One of the risks of working in such a large consortium, with several projects reliant on progress further upstream, is that delays in one area of the project can significantly impact longer term objectives of the project. A major benefit of installing such a pervasive system of oversight over the data was that such delays, were identified early on. This created a level of agility and adaptability that allowed consortium-level decisions to be made in a timely fashion.

### Accelerating research

Experience of the system has demonstrated that the data access policy has not unduly hindered or delayed access to study data. Indeed, the procedures devised have helped ensure a discipline in framing research questions and careful consideration by requestors of the data to which they require access. Delays, when they have occurred, have rather been because of the need to hold all data on a secure remote server with the associated precautions needed to ensure only legitimate access. A major advantage with DIRECT has been the long-term approach taken to planning research analysis – a reflection of the interlinking nature of many of the studies within DIRECT, and the ability to put together Analysis Plans in preparation for any data being made available. This greatly assisted the speed with which requests could be considered and ensured that requestors provided all relevant information with time for queries and discussions, as necessary.

One approach to ensure research objectives were met would be to let all researchers have access to all data, using an open access model (this could either be open to everyone, including outside of the consortium, or open to researchers within the consortium). However this approach would make it more difficult to maintain oversight to support continued collaborative working; it might engender competition rather than collaboration between partners, and thus discourage cross-utilisation of skills, which would undermine the value of bringing together a diverse range of partners. As mentioned above, the choice of data access governance was informed by the objectives of the project, and including as described to participants at recruitment: better prevention and clinical management of the disease from which they suffered. We found both data security and consortium efficiency were maximised by a controlled, but agile internal governance system. Our experience in DIRECT challenges whether this is the appropriate way to balance privacy and the Open Science agendas.

## Conclusion

In the DIRECT project, a governance system for data access was put in place that was intended to provide researchers across disciplines and sectors with fair access and ensure that consortium members had the opportunity to pursue their research interests for the benefit of the consortium, while guarding against internal competition, or duplication of effort. The governance mechanisms were devised in accordance with the objectives of the project and the objectives of data subjects who provide data and samples for medical research. It sought to maximize the ethical use of data, and enable progress against project deliverables, in line with the Grant agreement between funders and partners. There are multiple challenges when working in collaboration. It is important that the internal workings of the consortium do not exacerbate any concerns or fears that individual researchers may experience about being side-lined, and instead must provide an infrastructure that allows the advantages of multidisciplinary team-working, and advances in innovation, to be realised.

By installing a centralised (and closed) system for requesting access to data, the DIRECT project teams from academia and industry, working in similar areas and asking related questions, could be matched up and encouraged to work together. The requirement for analysts to provide a clear analysis plan encouraged high-quality research, while allowing all parties in a particular area of research to be included if they wished. It also set out a clear understanding among members of analysis teams what was expected of them.

The purpose of this system was to build an arena to identify, review and solve problems, with a single point of contact for researchers to address any specific queries or concerns that they had, or to identify potential blind spots within the system. The DAC received several requests from analysts and research teams to solve specific issues locally. The opportunity for participants to be able to keep track of the data for which they are a custodian and have greater control over has been recognised and welcomed. Through the committees, this governance system also provided a mechanism for reflection and review of project policies, to make sure that they continued to be fit for purpose as the project developed. While the system was tailored specifically to the DIRECT project, many of the overriding objectives may be applicable to other large-scale consortia, working across sectors and national boundaries. The value of encouraging close collaboration has been demonstrated in the productivity of the consortium and the strong working relationships that have been established across teams. Finally, through the success in implementing this Data Access system, in terms of both compliance and efficiency, the value has been shown of considering the objectives of a research consortium when devising Data Access governance systems – in this case balancing data security and data access to fulfil the consortium objective of identifying diabetes biomarkers to optimise treatment.

## Abbreviations

CSV: comma-separated values
DAC: Data Access Committee
DAF: Data Access Form
DIRECT: DIabetes REsearCh on patient stratification
DTA: data transfer agreement
DTU: Technical University Denmark
eCRF: electronic case report form
ELR: ethical and legal requirements
ENGAGE: European Network for Genetic and Genomic Epidemiology
ICGC: International Cancer Genome Consortium
IMI JU: Innovative Medicines Initiative Joint Undertaking
SDC: Samples and Data Committee
SUMMIT: SUrrogate markers for Micro- and Macro-vascular hard endpoints for Innovative diabetes Tools
UK10K: Project looking at Rare Genetic Variants in Health and Disease
WP: work package
